# Deciphering the brain-gut axis: elucidating the link between cerebral cortex structures and functional gastrointestinal disorders via integrated Mendelian randomization

**DOI:** 10.3389/fnins.2024.1398412

**Published:** 2024-05-22

**Authors:** Zhiwei Xu, Fenglan Ning, Xuecheng Zhang, Qi Wang, Yimei Zhang, Yiting Guo, Hongling Jia

**Affiliations:** ^1^School of Acupuncture and Tuina, Shandong University of Traditional Chinese Medicine, Jinan, Shandong, China; ^2^Department of Acupuncture and Rehabilitation, Longkou Traditional Chinese Medicine Hospital, Yantai, Shandong, China; ^3^Department of Proctology, China-Japan Friendship Hospital, Beijing, China; ^4^Department of Acupuncture, Second Affiliated Hospital of Shandong University of Traditional Chinese Medicine, Jinan, Shandong, China; ^5^Department of Radiology, Second Affiliated Hospital of Shandong University of Traditional Chinese Medicine, Jinan, Shandong, China

**Keywords:** functional gastrointestinal disorders, cerebral cortex, Mendelian randomization, mediation analysis, anxiety, depression

## Abstract

**Background:**

Observational studies have suggested associations between functional gastrointestinal disorders (FGIDs) and variations in the cerebral cortex. However, the causality of these relationships remains unclear, confounded by anxiety and depression. To clarify these causal relationships and explore the mediating roles of anxiety and depression, we applied univariate, multivariable, and mediation Mendelian randomization (MR) analyses.

**Method:**

We utilized genome-wide association study (GWAS) summary data from the FinnGen database and the ENIGMA consortium, identifying genetic variants associated with irritable bowel syndrome (IBS), functional dyspepsia (FD), and cerebral cortex structures. Data on anxiety and depression came from FinnGen and a large meta-analysis. Utilizing a bidirectional univariate MR approach, we explored correlations between FD, IBS, and cortex variations. Then, independent effects were assessed through multivariable MR. A meta-analysis of these results, incorporating data from two cohorts, aimed to increase precision. We also explored the potential mediating roles of anxiety and depression.

**Results:**

Our findings indicate a negative causal correlation between FD and the thickness of the rostral anterior cingulate cortex (rACC) across both global and regional adjustments (β = −0.142, 95% confidence interval (CI): −0.209 to-0.074, *P.FDR* = 0.004; β = −0.112, 95%CI: −0.163 to-0.006, *P.FDR* = 0.003) and a positive causal correlation with the globally adjusted thickness of the superior frontal gyrus (SFG) (β = 0.107, 95%CI: 0.062 to 0.153, *P.FDR* = 0.001). The causal correlation with the rACC persisted after multiple variable adjustments (β = −0.137, 95% CI: −0.187 to-0.087, *P.FDR* = 1.81 × 10^−5^; β = −0.109, 95%CI: −0.158 to-0.06, *P.FDR* = 0.002). A significant causal association was found between globally adjusted surface area of the caudal anterior cingulate cortex (cACC) and IBS (odds ratio = 1.267, 95%CI: 1.128 to 1.424, *P.FDR* = 0.02). The analysis showed that neither anxiety nor depression mediated the relationship between FGIDs and cerebral cortex structures.

**Conclusion:**

Our research provides significant MR evidence of a bidirectional causal relationship between FGIDs and the cerebral cortex structures. This evidence not only confirms the two-way communication along the brain-gut axis but also illuminates the underlying pathophysiology, paving the way for identifying potential therapeutic approaches.

## 1 Introduction

Irritable bowel syndrome (IBS) and functional dyspepsia (FD) are common functional gastrointestinal disorders (FGIDs) (Ford et al., 2020a,b), now understood as resulting from complex gut-brain interactions (Drossman and Hasler, 2016). These disorders are prevalent, with nearly 40% of the general population fulfilling the diagnostic criteria for FGIDs (Sperber et al., 2021), and FD and IBS specifically affecting about 16 and 11.2% of people, respectively (Lovell and Ford, 2012; Ford et al., 2020a). The economic impact of these disorders is substantial. The direct nursing costs of IBS in various nations range from 1.5 to 4.3 billion dollars (Müller-Lissner and Pirk, 2002; Zhang et al., 2016; Goodoory et al., 2022), and the combined direct and indirect economic losses due to FD in the U.S. reach approximately 18 billion dollars annually (Lacy et al., 2013). Moreover, the overlap of FD and IBS symptoms, along with their psychosomatic comorbidities, aggravates the economic burden significantly (Huang et al., 2023; Kamp et al., 2023; Staudacher et al., 2023). Furthermore, these disorders also have a profound impact on quality of life, often leading to reduced work capacity and unnecessary surgeries due to misdiagnoses, thereby posing challenges to healthcare and society (Drossman et al., 1993; Buono et al., 2017; Aziz et al., 2018; Black et al., 2020).

Despite their widespread occurrence, FGIDs suffer from a lack of dedicated research funding and pose diagnostic challenges due to their subjective symptoms and the absence of visible structural changes (Mawe and Hoffman, 2013). The gut-brain axis plays a pivotal role in the pathophysiology of FGIDs, with recent research focusing on alterations in the brain associated with these disorders to uncover potential treatment avenues. Recent observational studies have identified cerebral cortex structure changes in patients with FGIDs, offering insights into potential pathophysiology and treatment approaches (Tillisch et al., 2011; Mao et al., 2023). In FD patients, a reduction in the medial prefrontal cortex and anterior cingulate cortex is evident, whereas discrepancies are noted in areas such as the mid cingulate cortex, left orbitofrontal cortex, and posterior cingulate cortex (Zeng et al., 2013; Liu et al., 2018). For IBS patients, there have been increases or decreases in extensive brain regions of the frontal and parietal lobes, but a consensus has not yet been reached (Yu et al., 2022). However, interpreting these observational correlations as evidence of a causal relationship between FGID and brain structure relies on several unstable and potentially unreliable assumptions, including the absence of unmeasured confounders and reverse causality (Seyedsalehi et al., 2023). The association between FGID and brain structural indicators may be confounded by early life factors such as socioeconomic status (Jednoróg et al., 2012), insomnia (Chen et al., 2023), etc. Furthermore, even if observational studies accurately reflect a causal association, the direction of this causality remains undetermined (Davey Smith and Hemani, 2014). The additional layer of complexity from co-occurring mental health conditions like anxiety and depression further complicates understanding the link between cerebral cortex structures and FGIDs (Koloski et al., 2012; Qi et al., 2016).

**Figure 1:**
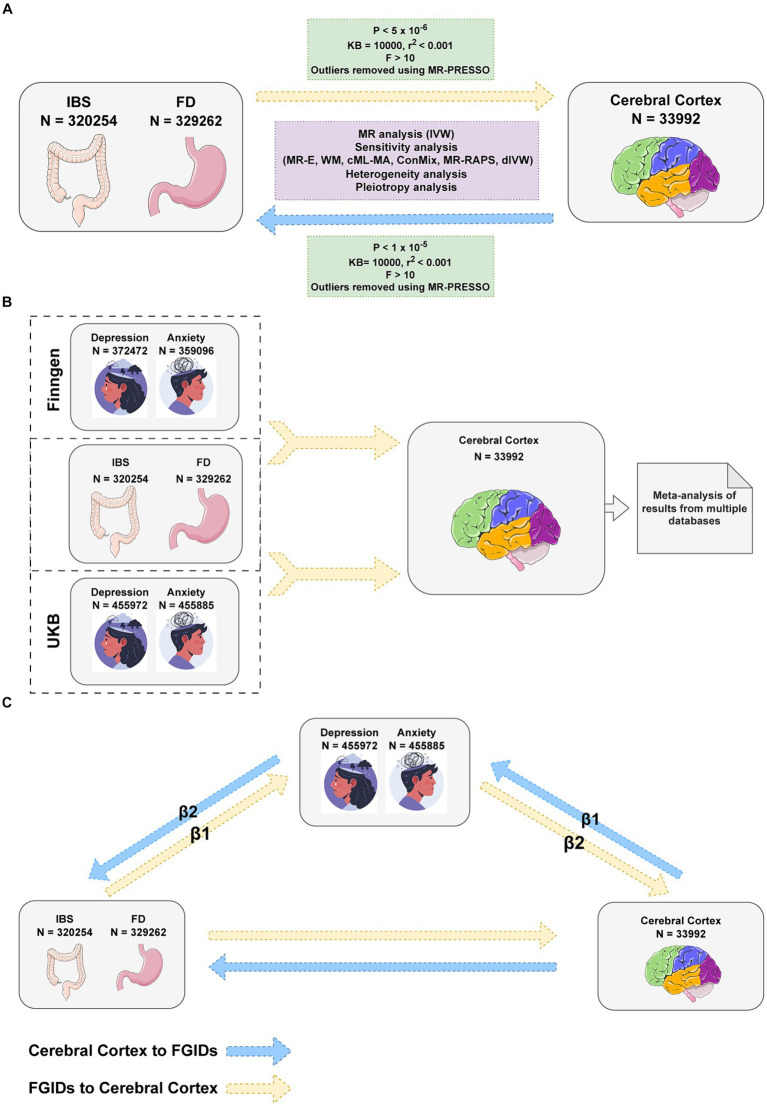
Flowchart of Mendelian randomization analysis. **(A)** Univariable Mendelian Randomization (MR) Analysis investigates the bidirectional causality between Functional Gastrointestinal Disorders (FGIDs) and cerebral cortical structure. **(B)** Multivariable MR Analysis adjusts for anxiety and depression and another FGID, considering the impacts of multiple exposures on the cerebral cortical structure. **(C)** Mediation MR Analysis explores the mediating roles of anxiety and depression. IBS, Irritable Bowel Syndrome; FD, Functional Dyspepsia; IVW, Inverse Variance Weighted method; MR-E, MR-Egger method; WM, Weighted Median method; MR-PRESSO, Mendelian Randomization Pleiotropy RESidual Sum and Outlier test; cML-MA, constrained maximum likelihood and model averaging method; ConMix, contamination mixture method; MR-RAPS, robust adjusted profile score method; dIVW, debiased inverse-variance weighted method. Images reproduced from Freepik and from Servier Medical Art. Images from Servier Medical Art are licensed under a Creative Commons Attribution 3.0 Unported License.

This study utilized genome-wide association study (GWAS) summary data on brain structure from Magnetic Resonance Imaging (MRI) and employed Mendelian randomization (MR) methods to investigate the gut-brain interaction in FGIDs. MRI plays a crucial role in exploring the gut-brain axis, allowing for a deeper understanding of these interactions in both health and disease (Mayer et al., 2019; Labus et al., 2023). MR, leveraging genetic variations as instrumental variables, aims to infer causality while minimizing bias from reverse causality and confounding (Burgess et al., 2013). Through a two-sample MR analysis of cerebral cortical structure MRI data from the ENIGMA consortium (Grasby et al., 2020), this study investigated the link between FGIDs and cerebral cortex structure. Additionally, considering the frequent co-occurrence of anxiety and depression with FGIDs, a multivariable Mendelian randomization (MVMR) approach was applied to adjust for these factors (Burgess and Thompson, 2015), with mediation MR employed to assess the potential mediating role of these mental health conditions in the relationship between cortical alterations and FGIDs (Figure 1).

## 2 Method

### 2.1 Three assumptions

MR analysis is grounded in three critical assumptions to validate its results: relevance, independence, and exclusion restriction (Davies et al., 2018). The relevance assumption mandates a robust association between the instrumental variables (IVs) and the exposure, typically validated by a *p*-value below 5×10^−8^. This ensures that the chosen IVs have a significant effect on the exposure of interest. The independence assumption necessitates that the IVs are not correlated with any confounders, guaranteeing that their relationship with the exposure is not influenced by external factors. This is essential for the IVs to reliably isolate the effects of the exposure. Lastly, the exclusion restriction criterion asserts that the IVs impact the outcome exclusively through their effect on the exposure, without any other alternative pathways. This condition is vital for correctly attributing observed effects to the exposure. Together, these foundational assumptions enable MR to effectively mitigate confounding influences, offering a more accurate estimation of the causal relationship between the exposure and the outcome.

### 2.2 Data source

The GWAS summary data for FD and IBS were sourced from the FinnGen database (Kurki et al., 2023), a collaboration of Finnish biobanks with health records from national registries. It comprises resources from nine biobanks, research institutions, hospitals, international pharmaceutical partners, and FINBB, with 412,000 samples as of August 2020 (Kurki et al., 2023), targeting half a million participants. The database is pivotal for uncovering genetic underpinnings of diseases and conducting GWAS on numerous health endpoints. Our FD study included 8,875 cases and 320,387 controls, while the IBS study comprised 9,323 cases and 301,931 controls.

The GWAS summary data on cerebral cortical structure were derived from a comprehensive meta-analysis conducted by the ENIGMA consortium (Grasby et al., 2020), which collated cortical measure studies from 51,665 individuals (94% of whom are European descendants) across 60 cohorts. The analysis focused on cortical surface area (SA) and thickness (TH), spanning total SA, mean TH, and both metrics across 34 functional cerebral cortex regions. The primary GWAS for these regions included total SA or mean TH as covariates, delineating region-specific genetic influences. Our aggregated analysis incorporated results from 33,992 individuals of European descent, examining a total of 138 phenotypes related to total SA, TH, and regional measurements, with both non-globally and globally adjusted outcomes.

For anxiety and depression, the GWAS summary data were bifurcated. The first segment originated from the FinnGen database, including 43,280 depression cases and 329,192 controls, alongside 21,519 anxiety cases with 337,577 controls. The complementary segment came from a large-scale meta-analysis utilizing the UK Biobank (UKB) database, incorporating 455,885 samples for anxiety and 455,972 for depression (Jiang et al., 2021). Further details on the GWAS datasets are provided in Supplementary Tables S1, S2.

### 2.3 Selection of genetic instruments

In our MR analysis, we selected genetic variations as IVs, meticulously following the core principles of relevance, independence, and exclusion restriction (Davies et al., 2018). Traditionally, MR analysis adopts a *p* value threshold of 5×10^−8^ to identify significant single nucleotide polymorphisms (SNPs). However, given the unique characteristics of our FGIDs dataset, we found no SNPs that reached significance at this level. Consequently, we adjusted our significance threshold to 5×10^−6^, and for the cerebral cortex study, we further relaxed it to 1×10^−5^ to accommodate the challenges posed by smaller sample sizes (Liu et al., 2023; Wang et al., 2023). To manage linkage disequilibrium (LD), we implemented a strict criterion of r^2^ < 0.001 across a 10,000 kb window, using European samples from the 1,000 Genomes Project as a reference. For the identification and correction of horizontal pleiotropy, we utilized the Mendelian Randomization Pleiotropy RESidual Sum and Outlier (MR-PRESSO) test to detect and remove outliers. Furthermore, to ascertain the robustness of each IV, we calculated the F-statistic (β^2^/se^2^) for every IV (Li et al., 2023), excluding any with an F-statistic less than 10. This process helped ensure the reliability of our IVs, thereby strengthening the validity of our MR analysis findings (Burgess and Thompson, 2011).

### 2.4 Mendelian randomization analyses

Our univariate MR analysis utilized the random-effects inverse variance weighted (IVW) method as our primary approach. This method assigns inverse variance as weights and is most effective when all IVs are valid (Hemani et al., 2018). To enhance the robustness of our findings, we employed several additional methods for sensitivity analysis. The Weighted Median method, akin to IVW but utilizing the median rather than the mean, ensures consistent estimates provided that at least 50% of the genetic variants are valid IVs (Bowden et al., 2016). The MR-Egger method introduces an intercept to account for horizontal pleiotropy, offering a valid test for the null hypothesis of no causal relationship even in the absence of effective IVs (Bowden et al., 2015). Additionally, the Contamination Mixture (ConMix) method employs maximum likelihood estimation to assess the probability of each genetic variant as an effective IV, yielding estimates with reduced variance (Burgess et al., 2020). The Constrained Maximum Likelihood and Model Averaging (cML-MA) method, based on constrained maximum likelihood, effectively identifies ineffective IVs, offering reliable causal effect estimates regardless of pleiotropic effects’ relevance (Xue et al., 2021). Furthermore, the debiased IVW (DIVW) method improves upon IVW by incorporating a bias correction factor, enhancing its robustness against weak IVs (Ye et al., 2021). Lastly, the Mendelian Randomization Robust Adjusted Profile Score (MR-RAPS) method specifically addresses both systematic and specific pleiotropic effects, ensuring accurate MR analyses with many weak instruments (Zhao et al., 2020).

We evaluated heterogeneity using the Cochran Q test, with a *p* value below 0.05 indicating significant heterogeneity (Greco et al., 2015). To address pleiotropy, the MR-PRESSO test was employed, complemented by the MR-Egger intercept test for further analysis (Bowden et al., 2016; Verbanck et al., 2018). Radial MR facilitated outlier detection and reevaluation (Bowden et al., 2018). The stability of results was confirmed through a leave-one-out analysis, systematically excluding each SNP in turn and recalculating the outcomes to ensure the robustness of our findings (Hemani et al., 2018).

Our MVMR analysis primarily utilized the multivariable IVW method (Burgess et al., 2015), with multivariable MR-Egger and Weighted Median methods as secondary approaches for sensitivity analysis. Anxiety, depression, and another FGIDs were incorporated as covariates, and then analyzed using a meta-analysis for more precise estimates. In mediation MR, we assessed the relationship between exposure and mediator variables (β1) and the mediator to outcome relationship (β2), quantifying the combined mediation effect as β1 x β2, evaluated using the Sobel test.

The overall workflow of our study is depicted in Figure 1. We adopted the false discovery rate (FDR) correction method to mitigate Type I errors. In our research, a post-FDR correction *p* value (*P.FDR*) below 0.05 is considered to indicate a significant association. When the *P.FDR* exceeds 0.05 but the original *p* value is below 0.05, it is classified as a nominally significant association. All statistical analyses and visualizations were conducted in R software (version 4.3.1), utilizing the Two SampleMR (version 0.0.7), Mendelian Randomization (version 0.9.0) (Yavorska and Burgess, 2017), RadialMR (version 1.1) (Bowden et al., 2018), forestploter (version 1.1.1), circlize (version 0.4.15) (Gu et al., 2014) and ComplexHeatmap (version 2.15.4) (Gu et al., 2016) packages.

## 3 Results

### 3.1 Selection of genetic instruments

In our univariate MR analysis, we identified 10 SNPs associated with FD and an additional 11 SNPs to explore the genetic connections to IBS. For our reverse MR analysis, we utilized 47 SNPs linked to cortical surface area and 39 SNPs associated with cortical thickness, along with a diverse range of 7 to 62 SNPs for examining the surface area and thickness of various functional areas of the brain. All chosen SNPs exhibited *F*-statistic values greater than 10, ensuring their robustness and affirming their validity as IVs. In our multivariable MR analysis, we identified 33 or 37 SNPs, each showing a strong correlation with at least one of the exposure factors being studied. To facilitate the mediation analysis, we pinpointed 7 to 10 SNPs that accurately represent the genetic influences on anxiety and depression. Further details regarding these SNPs can be found in Supplementary Table S3.

### 3.2 Causal effects of FGIDs on cerebral cortex

**Figure 2:**
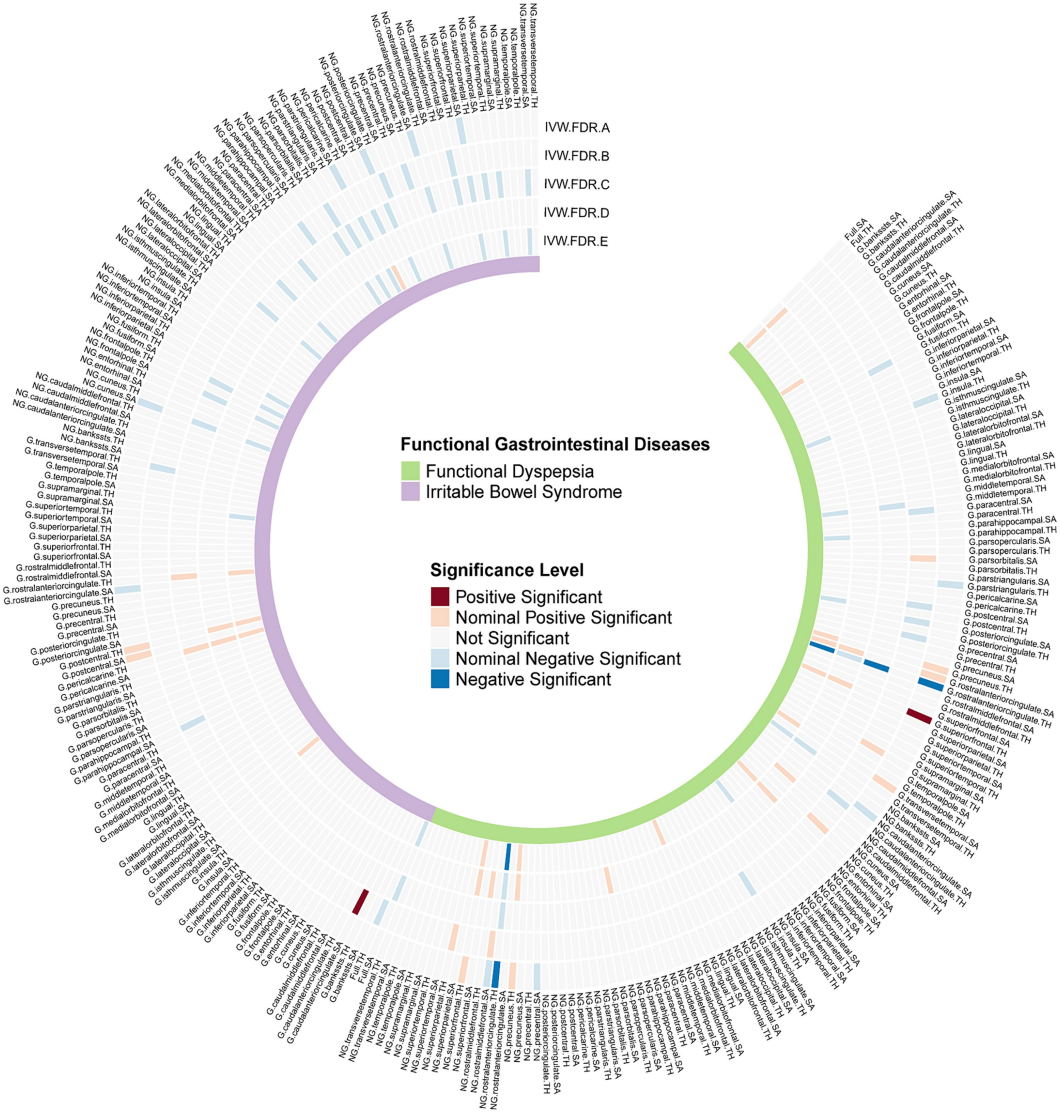
FDR-corrected **p** values of IVW results from all Mendelian randomization analyses. G, With globally adjusted; NG, With non-globally adjusted; SA, surface area; TH, thickness; IVW.FDR.A, Causal effects of FGIDs on cerebral cortex; IVW.FDR.B, Causal effects of cerebral cortex on FGIDs; IVW.FDR.C, Multivariable MR analyses adjusting depression and anxiety in FinnGen database; IVW.FDR.D, Multivariable MR analyses adjusting depression and anxiety in UKB database; IVW.FDR.E, Meta analysis for Multivariable MR analyses results.

We identified several significant and nominally significant associations (Figure 2). Notably, FD was inversely associated with the globally adjusted TH of the rostral anterior cingulate cortex (rACC) (β = −0.142, 95%CI: −0.209 to-0.074, *P.FDR* = 0.0036) and the non-globally adjusted TH of the rACC (β = −0.112, 95%CI: −0.163 to-0.06, *P.FDR* = 0.0031) (Table 1). A significant positive correlation was also found between FD and the globally adjusted average TH of the superior frontal gyrus (SFG) (β = 0.107, 95%CI: 0.062 to 0.153, *P.FDR* = 0.0012) (Table 1). After applying FDR correction, no significant associations were observed for IBS with any cerebral cortex structures. FD showed nominal associations with the globally adjusted SA of the rACC and the non-globally adjusted SA of the caudal anterior cingulate cortex (cACC), among others. IBS had nominal associations with areas including both the globally adjusted and the non-globally adjusted SA of the rACC, as well as the globally adjusted TH of the rACC. These associations are detailed in Supplementary Table S4.

**Table 1:** All significant univariate Mendelian randomization results and sensitivity analyses.

Exposure	Outcome	Method	Beta	95%CI	*p*-value
FD	Globally adjusted TH of the rACC	IVW	−0.142	(−0.209, −0.074)	<0.001
		MR Egger	−0.102	(−0.277, 0.073)	0.29
		Weighted median	−0.125	(−0.222, −0.027)	0.012
		Con Mix	−0.197	(−0.302, −0.092)	0.006
		RAPS	−0.145	(−0.221, −0.069)	<0.001
		dIVW	−0.148	(−0.22, −0.077)	<0.001
		cML-MA	−0.144	(−0.22, −0.069)	<0.001
FD	Globally adjusted TH of the SFG	IVW	0.107	(0.062, 0.153)	<0.001
		MR Egger	0.055	(−0.107, 0.216)	0.54
		Weighted median	0.102	(0.008, 0.196)	0.034
		Con Mix	0.116	(0.016, 0.216)	0.011
		RAPS	0.109	(0.026, 0.192)	0.01
		dIVW	0.112	(0.033, 0.192)	0.006
		cML-MA	0.108	(0.029, 0.187)	0.007
FD	Non-globally adjusted TH of the rACC	IVW	−0.112	(−0.163, −0.06)	<0.001
		MR Egger	−0.071	(−0.231, 0.09)	0.42
		Weighted median	−0.124	(−0.213, −0.035)	0.006
		Con Mix	−0.149	(−0.239, −0.059)	0.007
		RAPS	−0.114	(−0.186, −0.041)	0.002
		dIVW	−0.117	(−0.187, −0.046)	0.001
		cML-MA	−0.113	(−0.186, −0.04)	0.003
Globally adjusted SA of the cACC	IBS	IVW	0.237	(0.12, 0.353)	<0.001
		MR Egger	0.268	(−0.088, 0.624)	0.16
		Weighted median	0.267	(0.047, 0.487)	0.017
		Con Mix	0.372	(0.162, 0.582)	0.012
		RAPS	0.246	(0.078, 0.413)	0.004
		dIVW	0.246	(0.084, 0.409)	0.003
		cML-MA	0.235	(0.044, 0.426)	0.016

FD, functional dyspepsia; TH, thickness; IBS, irritable bowel syndrome; SA, surface area; rACC, rostral anterior cingulate cortex; SFG, superior frontal gyrus; cACC, caudal anterior cingulate cortex; IVW, Inverse Variance Weighted method; MR-E, MR-Egger method; WM, Weighted Median method; MR-PRESSO, Mendelian Randomization Pleiotropy RESidual Sum and Outlier test; cML-MA, constrained maximum likelihood and model averaging method; ConMix, contamination mixture method; MR-RAPS, robust adjusted profile score method; dIVW, debiased inverse-variance weighted method.

In our analysis of the three significant correlations associated with FD, we found consistent and robust results. Initially, the Cochran Q test showed no significant heterogeneity among the studies (Supplementary Table S4), indicating that our findings are reliable across different genetic variants. Similarly, the MR-Egger method revealed no evidence of significant horizontal pleiotropy (Supplementary Table S4), suggesting that the genetic variants used as instruments affect the outcome primarily through their impact on FD, rather than through other pathways. This conclusion is further supported by the funnel plot, which exhibited no asymmetry (Supplementary Figure S1), and the leave-one-out analysis, which identified no significant outliers (Supplementary Figure S2), both affirming the robustness of our results. Moreover, although the confidence interval of MR-Egger includes zero, the direction of the point estimate aligns with that obtained using the IVW method (Table 1), reinforcing the consistency of our findings. The point estimates from other robustness methods not only share this direction but are also statistically significant (Table 1), underscoring the strength of the association between the genetic instruments and FD. For comprehensive details on these analyses, refer to Supplementary Table S4.

### 3.3 Multivariable MR analyses adjusting depression, anxiety, and another FGID

In our multivariable MR analysis, we incorporated anxiety and depression GWAS summary data from FinnGen and UKB as covariates. This approach allowed us to accurately assess the correlation between FGIDs and cerebral cortex structures while controlling for anxiety, depression, and other FGIDs. Utilizing a random-effects meta-analysis model, we aggregated the estimates and observed several notable associations (Figure 2).

**Figure 3:**
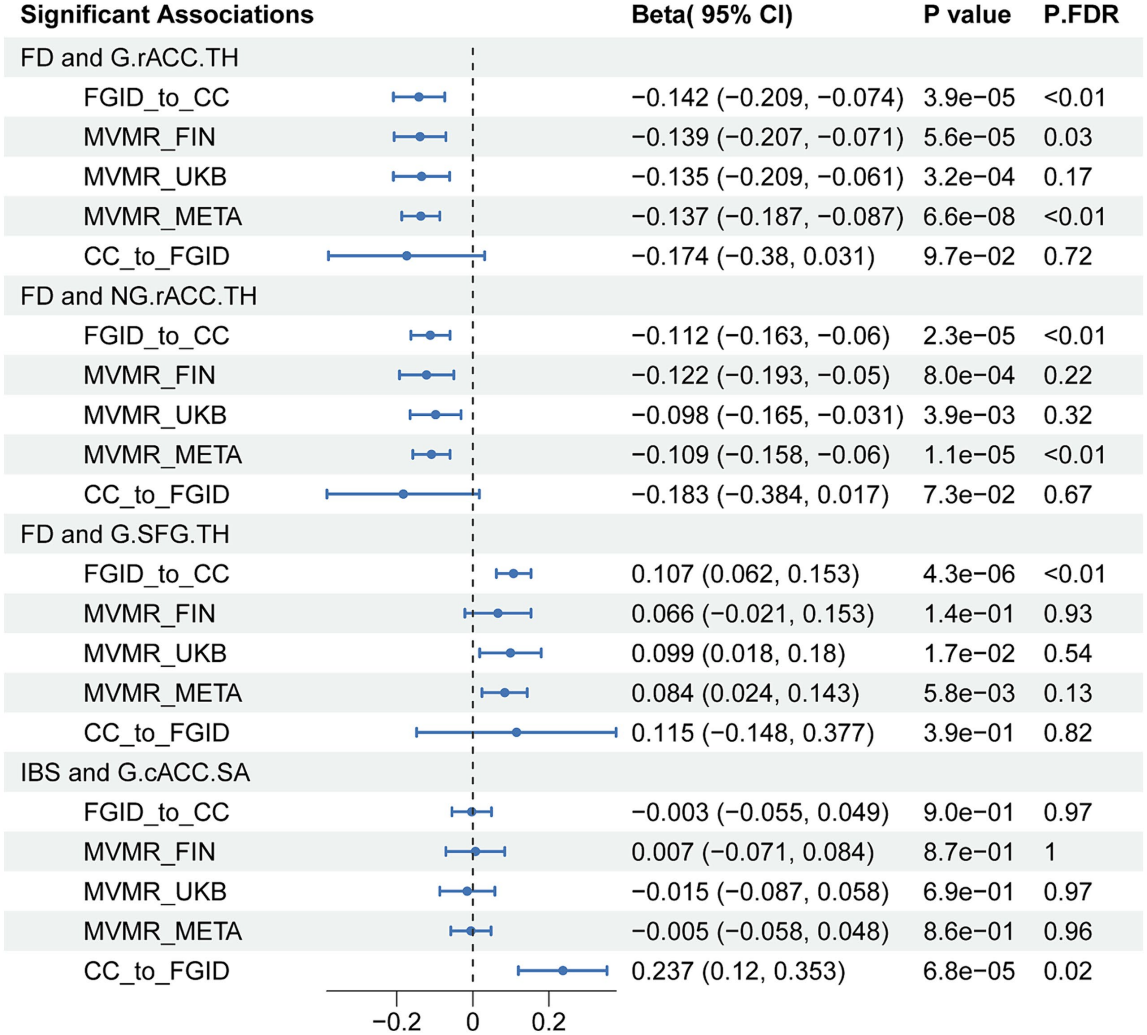
IVW results of all significant associations from Mendelian randomization analyses. FD, functional dyspepsia; IBS; Irritable bowel syndrome; CC, cerebral cortex; G, globally adjusted; NG, non-globally adjusted; SA, surface area; TH, thickness; FGID, functional gastrointestinal disorders; MVMR, multivariable Mendelian randomization; P.FDR, FDR-Corrected *p* values of IVW.

We found that the negative correlation between FD and the TH of the rACC remained after adjusting for anxiety, depression, and IBS in both globally and non-globally adjusted measurements (β = −0.137, 95%CI = −0.187 to-0.087, *P.FDR* = 1.81 × 10^−5^; and β = −0.109, 95%CI = −0.158 to-0.060, *P.FDR* = 0.0015) (Figure 3). However, the association between FD and the globally adjusted TH of the SFG became only nominally significant after adjusting for FDR. Interestingly, some correlations that were not nominally significant in the univariate analysis gained nominal significance in the multivariable analysis, particularly the relationship between FD and the full TH (β = 0.072, 95%CI: 0.015 to 0.128, *p* = 0.013), and between IBS and the full SA (β = −0.082, 95%CI: −0.152 to-0.012, *p* = 0.022).

In our approach to addressing the statistical heterogeneity noted in several associations, we implemented the random-effects multivariable IVW method. This technique compensates for variations across studies, delivering more cautious and inclusive estimations of effect sizes. Additionally, to evaluate the potential for pleiotropy, we utilized the MR-Egger intercept test, which disclosed no significant pleiotropic effects within our principal findings. The alignment of estimates obtained from three distinct statistical methodologies further solidified the reliability of our conclusions. More comprehensive results of the MVMR and meta-analysis are detailed in Supplementary Tables S5–S7.

### 3.4 Causal effects of cerebral cortex on FGIDs

In the reverse MR analysis, we aimed to investigate the potential causal effects of cerebral cortical structure on FGIDs (Figure 2). We found a significant positive causal correlation between the globally adjusted average SA of the cACC and IBS, with an odds ratio (OR) of 1.267 (95% CI: 1.128 to 1.424, *P.FDR* = 0.019) (Table 1). However, no association was found between the globally adjusted average SA of the cACC and IBS nor between any cerebral cortical structures and FD. Additionally, several nominally significant correlations were identified, including the association between the non-globally adjusted TH of the cACC and FD, and the correlation between full TH and IBS, among others.

Our sensitivity analyses indicate robust results with minimal influence from confounding factors. The Cochran Q test confirms the absence of significant heterogeneity across studies (Supplementary Table S8), ensuring the uniformity of effects observed in various environments. This finding is reinforced by the MR-Egger method, which shows no evidence of significant horizontal pleiotropy (Supplementary Table S8), indicating that the genetic variations used as instruments directly influence the outcome, not through other variables. The symmetry of the funnel plot (Supplementary Figure S1) and the lack of significant outliers in the leave-one-out analysis (Supplementary Figure S2) further validate the robustness of the results. Notably, the confidence interval of the MR-Egger method includes zero, but its point estimate direction is consistent with the IVW method (Table 1), highlighting the consistency of effect directions across methodologies. Furthermore, the point estimates from additional robustness analyses not only align in direction but are also statistically significant (Table 1), solidifying the credibility of our findings. Comprehensive details of these analyses are thoroughly documented in Supplementary Table S8 for in-depth review.

### 3.5 Causal effects of FGIDs on cerebral cortex mediated by anxiety and depression

To minimize the impact of sample overlap, we utilized data on anxiety and depression from the UK Biobank (UKB) as mediators in our mediation analysis. Our objective was to explore their roles in mediating the relationship between FGIDs and cerebral cortex structures. However, despite our comprehensive efforts, our analysis revealed no significant mediation effects of these psychological conditions on the relationship between FGIDs and the cerebral cortex. Similarly, when investigating the potential influence of cerebral cortex structure on FGIDs, with anxiety and depression again serving as mediators, we found no significant mediation effects. Table 2 presents a detailed breakdown of the mediation effects, which includes analyses of direct, indirect, and total impacts, alongside results from the Sobel test for thorough evaluation.

**Table 2:** Results of mediation effect analysis.

Exposure	Mediator	Outcome	Total effect	Mediating effect	Mediating effect ratio	P value of sobel test
FD	Anxiety	Globally adjusted TH of the rACC	−0.142 (0.209, −0.074)	0.003 (−0.007, 0.014)	−2.15%	0.55
FD	Anxiety	Non-globally adjusted TH of the rACC	−0.112 (−0.163, −0.060)	0.003 (−0.008, 0.014)	−2.91%	0.55
FD	Depression	Globally adjusted TH of the rACC	−0.142 (−0.209, −0.074)	0.004 (−0.008, −0.016)	−2.79%	0.49
FD	Depression	Non-globally adjusted TH of the rACC	−0.112 (−0.163, −0.060)	0.003 (−0.006, 0.010)	2.30%	0.50
Globally adjusted SA of the cACC	Anxiety	IBS	0.179 (0.056, 0.302)	0.001 (−0.008, 0.009)	0.33%	0.80
Globally adjusted SA of the cACC	Depression	IBS	0.179 (0.056, 0.302)	0.001 (−0.006, 0.009)	0.60%	0.70

FD, Functional dyspepsia; IBS, Irritable bowel syndrome, cACC, caudal anterior cingulate cortex, rACC, rostral anterior cingulate cortex.

## 4 Discussion

To our knowledge, this is the first large-sample MR study to explore the bidirectional causal relationship between cerebral cortex structures and FGIDs. Our robust approach included univariate, multivariable, and mediation analyses. Initially, univariate analysis revealed significant negative causal correlations between FD and the TH of rACC, both globally adjusted and non-globally adjusted, and a positive causal correlation with the globally adjusted TH of the SFG. A combined multivariable and meta-analysis later reinforced the significant negative correlation between FD and rACC thickness. Conversely, reverse MR analysis showed a significant positive causal link between the TH of cACC and IBS susceptibility. However, no significant mediating effects of anxiety or depression were found on the FD-cerebral cortex structures relationship. The gut-brain axis is integral to the development of FGIDs, involving alterations in brain function and increased mental health issues among patients with FGIDs (Koloski et al., 2016). Our comprehensive MR analysis sought to establish direct causality by accounting for these psychological factors. By focusing on biomarkers, our study provides avenues for employing these markers in diagnosis, treatment monitoring, and early intervention in FGID-related mental disorders.

The primary discovery reveals a significant association between FD and reduced cortical thickness in the rACC, suggesting a potential causal relationship. Neuroimaging research has increasingly focused on changes in brain function or structure associated with FD, with the rACC being of particular interest due to its central role in regulating emotions (Boes et al., 2018), pain perception (Sun et al., 2024), and cognitive functions (Wang et al., 2022). FD manifests as upper abdominal pain, a burning sensation, bloating, and early satiety, and involves increased gastrointestinal sensitivity (Enck et al., 2017). Notably, patients frequently report emotional symptoms such as anxiety and depression (Huang et al., 2023), which underscores the potential involvement of the gut-brain axis in FD.

Supporting this, our results are consistent with previous findings; for instance, a structural Magnetic Resonance Imaging (sMRI) study comparing 69 FD patients with 49 healthy controls (HC) highlighted reduced cortical thickness in several brain regions, including the ACC, among FD patients (Liu et al., 2018). This is significant despite the scarcity of research specifically on cortical thickness changes in FD, as existing studies document decreased grey matter density (GMD) in the ACC of FD patients (Zeng et al., 2013). Importantly, these neuroanatomical changes negatively correlate with the severity of FD symptoms, establishing a link between structural alterations in the brain and clinical manifestations of FD. deCharms et al. proposed that individuals might learn to modulate pain perception by controlling rACC activity (deCharms et al., 2005), suggesting that changes in the rACC could impair the endogenous pain regulation mechanism, leading to altered pain modulation. Studies have shown that chronic pain can lead to decreased brain grey matter (Rodriguez-Raecke et al., 2009), indicating that this reduction is more a consequence than a cause of pain, which begins to clarify the direction of causation. Further research indicates that a thicker rACC is associated with better emotional regulation (Wu et al., 2022), implying that FD patients with reduced rACC thickness may struggle with regulating emotional responses to negative or stressful stimuli, potentially explaining the high prevalence of psychological issues such as anxiety and depression in FD patients.

The continuous or repetitive upper abdominal pain and discomfort characteristic of FD may affect brain areas responsible for pain and emotional management, notably through the brain-gut axis interaction, with the rACC being a critical focus. Prolonged exposure to emotional stress and pain perception might lead to functional overactivity in the rACC (Zeng et al., 2011; Nan et al., 2015; Lee et al., 2016). We hypothesize that such excessive activation of the rACC over time could induce structural changes, including reduced cortical thickness. This brain adaptation, in response to ongoing emotional and pain stimuli from FD, reflects a form of neuroplasticity.

Our subsequent multivariable and mediation MR analysis indicates that the causal link between FD and reduced rACC thickness remains robust, even after adjusting for factors such as anxiety, depression, and IBS. Furthermore, anxiety and depression do not significantly mediate this causal pathway, reinforcing the notion of a direct link between FD and structural changes in the rACC, independent of the effects of anxiety and depression on cortical thickness. However, an observational study found that, after controlling for anxiety and depression, the difference in rACC cortical density between FD patients and healthy controls was not statistically significant (Zeng et al., 2013). This contrasts with the findings of MR and may highlight limitations of the observational study, such as potential confounders and measurement errors. The design principles and methodological strengths of MR lend its conclusions greater reliability, suggesting a solid scientific basis for a direct causal relationship between FD and changes in the rACC. The discrepancy between MR and observational studies suggests the need for future research using more rigorous designs, such as prospective cohort studies and detailed genetic analyses, to confirm our findings.

The interplay between FD and the rACC underscores a complex dynamic where the ongoing pathophysiological stimuli of FD could lead to structural changes in the rACC. These changes might exacerbate the clinical symptoms of FD by affecting mechanisms of pain and emotional processing. This insight underlines the importance of integrating psychosocial interventions and neuromodulation strategies into FD treatments and highlights the necessity of a comprehensive understanding of brain-gut interactions to develop holistic treatment approaches.

In the current study, another major finding is the establishment of a positive causal link between IBS and the surface area of the cACC for the first time, demonstrating that an increase in the surface area of the cACC elevates the risk of developing IBS. This finding is pivotal in understanding the brain-gut axis dysfunction, which is central to the pathogenesis of functional gastrointestinal disorders like IBS. By leveraging MR, the limitations of conventional observational studies were overcome, clarifying the causal direction by demonstrating that alterations in the surface area of the cACC precede the symptoms of IBS. This insight aligns with similar research findings (Roberts et al., 2022).

The cACC is crucial in pain processing (Trujillo et al., 2023), emotional regulation (Estévez-López et al., 2023), and cognitive functions (Wang et al., 2022; Xu et al., 2023), particularly in evaluating and responding to pain, which influences the subjective pain experience. Given the common reports of heightened abdominal pain sensitivity in IBS patients (Vasant et al., 2021), potentially due to brain processing anomalies, changes in the cACC could significantly affect perception of abdominal discomfort and emotional responses, influencing IBS onset and severity.

Research on structural changes in the cACC, especially its surface area, is limited. However, one study noted an increase in the gray matter density of the cACC in patients with IBS (Seminowicz et al., 2010), although most research has focused on functional activities (Wang et al., 2017). This gap allows us to propose that an enlarged cACC surface area might indicate hyperactive functionality, leading to an overactive pain response and dysfunctional emotional regulation. Such changes could amplify abdominal discomfort and disrupt gut-brain communication (Mertz et al., 2000; Van Oudenhove et al., 2007), thereby increasing IBS risk. Specifically, a larger surface area of the cACC could reflect intensified neural activity or connectivity (Ma et al., 2015), thereby enhancing sensitivity to gastrointestinal stimuli and altering the cognitive and emotional processing of pain. This could result in more intense or frequent abdominal pain and heightened emotional responses in IBS patients (Yu et al., 2022).

Our findings significantly contribute to the neurobiological understanding of IBS and to future therapeutic approaches. They highlight that structural changes in the cACC are a critical aspect of the pathophysiology of IBS and illuminate the condition’s complex biological mechanisms. Moreover, this discovery opens new treatment avenues. While current strategies focus on symptom management, our research suggests that targeting the ACC with specific interventions, such as neuromodulation or tailored psychotherapies, could alter the structure and function of the brain in patients with IBS, offering symptom relief.

This study has several strengths. We used an MR design to reduce bias and reverse causality. Our focus on a European population helped minimize stratification bias. We enhanced the reliability of our findings through robust sensitivity analyses and pleiotropy tests, using MR-PRESSO and RadialMR methods for detected pleiotropy. Causal relationships were established when all of the seven different analytical methods agreed. Our multivariable analyses accounted for potential confounders like anxiety, depression, and other FGID-related diseases, confirming our univariate results. We also examined the mediation effects of anxiety and depression on the FGIDs-cerebral cortex structures relationship, adding depth to our understanding of the underlying mechanisms.

This study acknowledges several limitations. Firstly, the assumption of a linear correlation between exposure and outcome in MR may not fully capture all nonlinear interactions. In addition to the primary analysis method of IVW, we employed six other robust methods, all providing directionally consistent causal estimates under the assumption of linearity. Moreover, the Cochran Q tests indicated good consistency among all instrumental variables, reinforcing the reliability of our linear model. Despite limitations related to data and model types, our results still demonstrate robustness. We acknowledge current skepticism towards nonlinear models (Wade et al., 2023); however, we believe these models hold potential for elucidating complex biological relationships. This encourages further validation of our study results with more detailed individual data and advanced nonlinear algorithms.

Secondly, the potential influence of unmeasured confounders persists despite our rigorous statistical controls. We adjusted for variables such as anxiety, depression, and another FGID, and applied pleiotropy tests like MR-Egger intercept test and MRPRESSO to assess and attempt to mitigate these confounders. The nonsignificant outcomes of these tests support the robustness of our causal link and enhance the credibility of our IVs. However, the possibility of slight interference from unmeasured confounders on the results cannot be entirely dismissed. Insomnia and gut microbiota in FGID and brain structure appear to play possible confounding roles. Recent studies have established causal relationships between insomnia, gut microbiota, and brain structure; however, these studies highlight specific areas affected such as the medial orbitofrontal cortex’s surface area and the average thickness of gray matter (Huang et al., 2023; Wang et al., 2023). These areas do not directly relate to structures such as the rACC and cACC implicated in our results, suggesting that these confounders likely do not impact our outcomes. Future research should focus on identifying and including additional confounders that may impact the brain-gut axis, employing more comprehensive multivariable models.

Thirdly, the study primarily focused on the structural aspects of the cerebral cortex and its interactions with FGIDs, representing only a segment of the multifaceted brain-gut axis. This axis encompasses multiple physiological pathways, including the autonomic nervous system (ANS), hypothalamic–pituitary–adrenal (HPA) axis, and enteric nervous system (ENS) (Ganci et al., 2019; Chen et al., 2022). To enhance our understanding of the brain-gut axis comprehensively, future studies should incorporate various modalities such as brain functionality, structural outcomes, and biochemistry (Mayer et al., 2019). Our current data limitations prevented a detailed examination of subcortical structures, overall brain functionality, and connectivity, which are essential for a thorough understanding of brain-gut interactions. Future research should employ both functional and structural imaging techniques and consider interdisciplinary approaches that integrate physiological, psychological, and molecular data.

Fourthly, the validity of certain IVs may still raise concerns. However, each of our IVs has an F-statistic of at least 19, indicating strong predictors of exposure and thus reducing the likelihood of imprecise estimates caused by weak IVs. We employed robust methods such as dIVW and MR RAPS, which are capable of tolerating biases introduced by some weak IVs (Zhao et al., 2020; Ye et al., 2021). The significant estimates from these methods suggest that the impact of such biases is minimal. Additionally, sensitivity analyses, including leave-one-out tests, have confirmed the robustness of our findings, showing that our results are not overly dependent on any single IV. Future research should continue to explore and validate the strength and appropriateness of IVs in MR studies, potentially incorporating new statistical techniques to more effectively address weak instruments and validate our findings in larger-scale genome-wide association studies.

Fifthly, the generalizability of our findings is limited, as the study sample was confined to a European population. Future research should extend to more diverse populations to enhance the external validity of the results.

Lastly, MR assumes that genetic variations are determined at conception, which may not fully account for the effects of somatic mutations.

## 5 Conclusion

Our research furnishes MR evidence supporting a bidirectional causal relationship between FGIDs and cerebral cortex structures, highlighting the critical role of the brain-gut axis in mediating this interaction. This discovery offers new insights into the pathophysiology, which could significantly influence future therapeutic strategies. Nevertheless, the inherent complexity and variability of the domain, further research is essential to elucidate the exact mechanisms underlying this relationship. Our findings make a substantial contribution to the field, potentially sparking further inquiries and laying the groundwork for continued scientific exploration.

## Data availability statement

The original contributions presented in the study are included in the article/Supplementary material, further inquiries can be directed to the corresponding author.

## Ethics statement

Ethical approval was not required for the studies involving humans because our study constitutes a secondary analysis based on publicly available data that has already received ethical review board approval. Therefore, an additional ethical review was not necessary for this research. The studies were conducted in accordance with the local legislation and institutional requirements. Written informed consent for participation was not required from the participants or the participants’ legal guardians/next of kin in accordance with the national legislation and institutional requirements because our study constitutes a secondary analysis based on publicly available data that has already received ethical review board approval.

## Author contributions

ZX: Conceptualization, Data curation, Formal analysis, Methodology, Software, Writing – original draft. FN: Data curation, Formal analysis, Methodology, Software, Validation, Visualization, Writing – original draft. XZ: Investigation, Visualization, Writing – original draft, Writing – review & editing. QW: Writing – review & editing. YZ: Writing – review & editing. YG: Conceptualization, Supervision, Writing – review & editing. HJ: Conceptualization, Supervision, Writing – review & editing.
