# Supplementary material for: Deciphering the brain-gut axis: elucidating the link between cerebral cortex structures and functional gastrointestinal disorders via integrated Mendelian randomization
Source: Front Neurosci. 2024 May 22;18:1398412. doi: 10.3389/fnins.2024.1398412 (PMC11152161; doi:10.3389/fnins.2024.1398412)
Supplement: Supplementary file 2 [file Data_Sheet_2.docx]

Supplementary Material

## Supplementary Figures


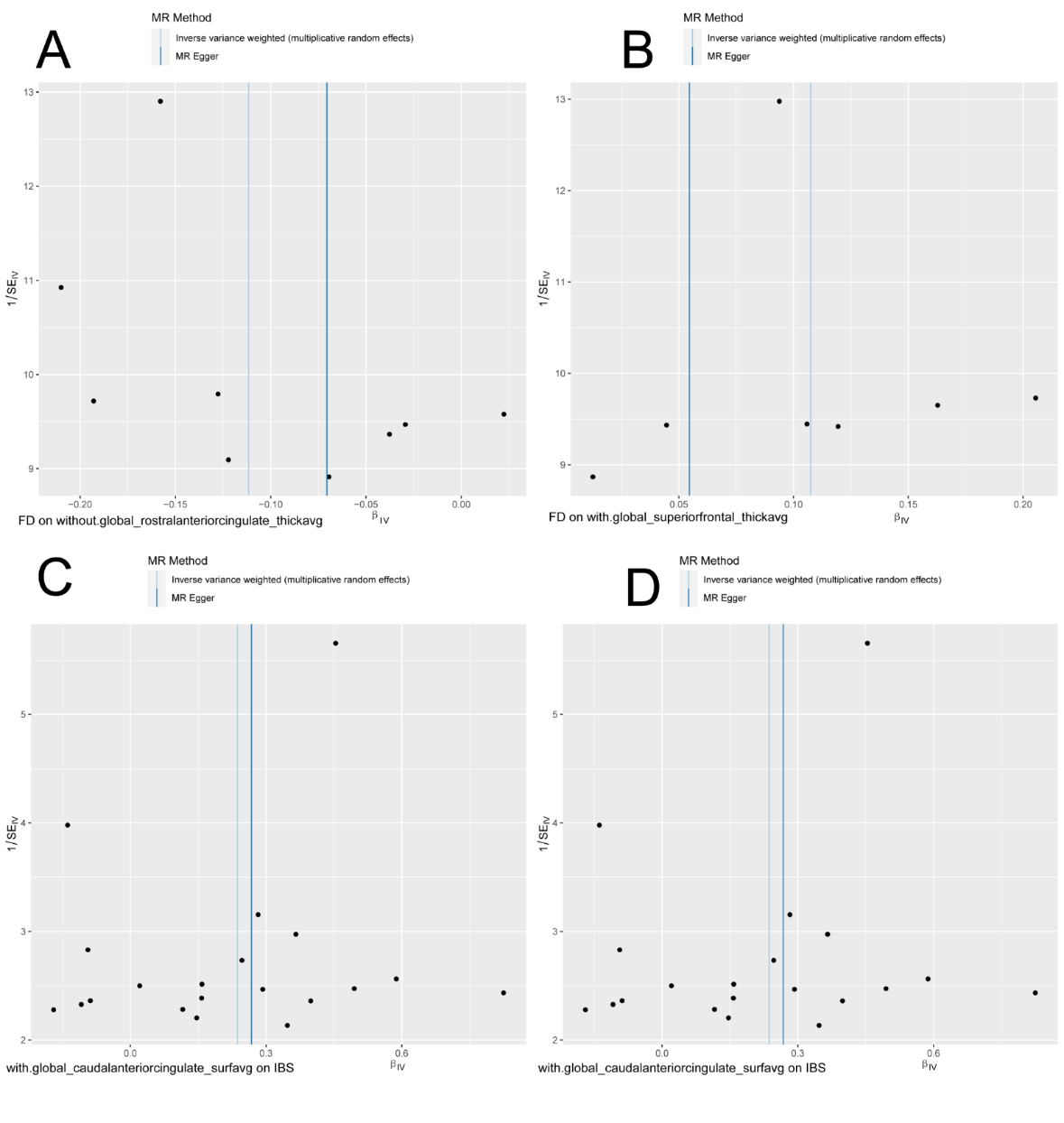


**Supplementary Figure 1. The funnel plot of all significant associations.** A: causal effects of functional dyspepsia (FD) on the thickness of the rostral anterior cingulate cortex (rACC) without global weighted; B: causal effects of functional dyspepsia (FD) on the thickness of the rostral anterior cingulate cortex (rACC) with global weighted; C: causal effects of FD on the thickness of the superior frontal gyrus with global weighted; D: causal effects of the surface area of the caudal anterior cingulate cortex with global weighted on irritable bowel syndrome.


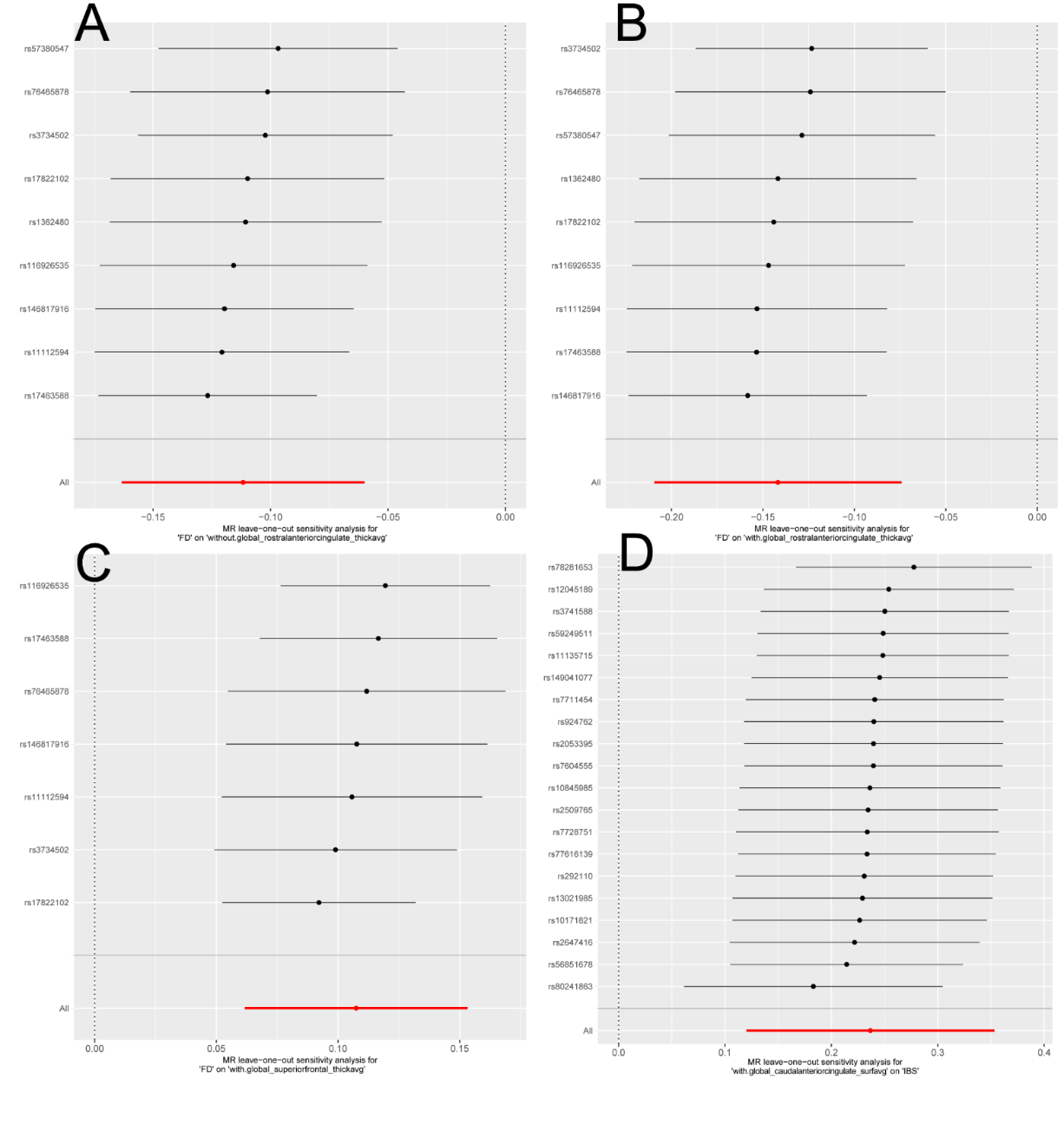


**Supplementary Figure 2.** The leave-one-out plot of all significant associations. A: causal effects of functional dyspepsia (FD) on the thickness of the rostral anterior cingulate cortex (rACC) without global weighted; B: causal effects of FD on the thickness of the rACC with global weighted; C: causal effects of FD on the thickness of the superior frontal gyrus with global weighted; D: causal effects of the surface area of the caudal anterior cingulate cortex with global weighted on irritable bowel syndrome
